# Editorial: Health Technologies and Innovations to Effectively Respond to the Covid-19 Pandemic

**DOI:** 10.3389/fdgth.2022.849652

**Published:** 2022-02-14

**Authors:** Pradeep Nair

**Affiliations:** Department of New Media, School of Journalism, Mass Communication & New Media, Central University of Himachal Pradesh, Dharamsala, India

**Keywords:** health technology, innovation, healthcare, digital health, COVID-19

Covid-pandemic has an unprecedented impact on global, regional, and national health systems across countries. Both public and private healthcare sectors had struggled and are still struggling to respond to the impact of the pandemic. The struggle is not only about adopting diverse healthcare responses in terms of cutting-edge technological tools and innovations in the areas of public health, medicine and wellness to take prompt decisions to address the pandemic by flattening the disease curve but also to revisit and reopen the realm of “digital health” in the policy and public discourse.

While keeping in view, the short, medium and long term response strategies, it is an appropriate time to take a tangible shift toward holistic technology and data-driven digital tools. This will help to engage both public and private healthcare systems across the country in facilitating policy dialogue, technical assistance and training on specialized policy and response interventions at the regional and national level. It is also a fact that the well-tested hardware are already in place and quite a few experiments have been conducted but has not been in good use, particularly in developing nations, for lack of national will, want of resources, and strategic planning to reach those who really need it most.

An alignment of expertise, leadership, and practices are required to synthesize knowledge and experience to assess capacities and avenues of emerging technologies such as artificial intelligence (AI), machine learning, Blockchain, health wearables, remote patient monitoring trackers, sensor-enabled hospital beds, medication-tracking systems and medical supplies and equipment inventory tracking systems. This will provide policy dialogue and responses to make the healthcare systems inclusive and accessible in terms of better patient experience and cost (1).

With the advent of newer technology, it will percolate down. The real concern is its application up to the last point. The first telemedicine experiments in developing nations were conducted way back in 1970s and 80s and a couple of more “resourceful” healthcare operators use it off and on since the early 1990s. The problem with the developing countries is to integrate the emerging healthcare technologies into the operational situation, that too for all. We should remember that any technology has the inherent characteristics of being used by those who can afford it. So, either it is a tool in the hand of private players (many of them have state of art technology) or the cost is subsumed by the State, which failed in the last so many decades to take to the last point. Educating and communicating people about the benefits of healthcare technology would come later when it is made available to them.

The potential of telemedicine is well observed during the pandemic time as several consultations with doctors/health experts happened without going to the clinic or hospital. This was a positive sign and urges to look into new business models to help further telemedicine across the world. On this front, it is a time to encourage companies to come to the forefront with a wide range of tech-driven digital healthcare tools and products. It was predicted by the health industry experts that tele-health market across the world is likely to witness a massive spike both from the demand and supply sides. The service providers are assessing capacities and avenues, pursuing evidence-based innovations and technologies across the board, including diagnostic and telemedicine tools, cellphone apps for fitness, well-being, medical and healthcare, and data-driven software. But the argument is that maybe it is the pandemic which compelled to explore and extend these technologies to deal with the spatial and temporal gaps to access healthcare. The concern shall be to look into how these technologies and innovations will be used in a post-covid world to deal with public health issues (2). How in coming days, technologies and innovations will be integrated with public health response system for greater accessibility and affordability to health care services should be a priority area of research in public health studies. The pandemic has unraveled the myriad avenues and opportunities in boosting healthcare and life sciences in the world, it would be interesting to look into how health shall be observed as wealth in the post Covid world with technology and innovations as its greatest investment.

The recent studies on Covid-19 have shown that the rates of severity of the impact of the pandemic are more on people from the less privileged communities. Medical devices and applications that are connected to healthcare IT systems via the internet known as “The Internet of Medical Things (IoMT)” has the potential to provide regular access to health care services through cloud-connected health care professionals. The concept of “data governance” in healthcare can offer safe and secure digital ecosystem to manage the handling, storing and sharing of real-time patient data 24 x 7 across hospitals and healthcare institutions to the next level with clearly defined policies and procedures.

This Research Topic collection attempted to explore the new post-pandemic health realities with a focus on various policy and response strategies spearheaded by the countries across the world to make public healthcare services more accessible to citizens in terms of cost, security and data privacy issues. The diverse studies published under the Research Topic collection looked into the new structural and institutional shifts taking place to make healthcare services safe and convenient with the help of empowered frontline care leveraging technologies. To make the healthcare services more inclusive and accessible, healthcare organizations and institutions, both public and private need to orchestrate the myriad interconnected changes required to design, implement and sustain digitally-enabled healthcare delivery platforms.

The Research Topic collection addressed various policy and response strategies adopted to deal with restricted physical access to socio-economic infrastructure, facilities and services amid the pandemic with a focus on cutting-edge health-technologies at its core. The topic collection strongly advocates that health technologies and innovations are going to be one of the significant sectors for investment and innovations over the next 30 years. This will not only transform the global health care sector in terms of diagnosis, disease management, treatment and prevention but also help to better prepare for future emergencies.

The published topic collection had studies having a diverse and vast coverage. The mini review “Covid-19 and Computer Audition: An overview on what speech and sound analysis could contribute in the SARS-CoV-2 Corona crisis” by Schuller et al. advocates the use of Computer Audition (CA) for implementation of (pre-) diagnosis and monitoring tools, and more generally provides rich and significant contribution in the fight against Covid-19 spread. A brief research report titled “Early warning signs of a mental health tsunami: A coordinated response to gather initial data insights from multiple digital services providers” by Becky Inkster and Digital Mental Health Data Insights Group (DMHDIG) provides evidence based concept for researchers and private companies to work collaboratively on a diverse range of mental health concerns. The research report observed that there is an increasing demand for digital mental health support during Covid due to an increased presentation of anxiety and loneliness. The opinion on “Out-of-Hospital care of heart failure patients during and after Covid-19 pandemic: Time for Telemedicine” by Faragli et al. talks about how telemedicine has turned as an essential requirement during covid time. The opinion piece strongly advocates the use of telemedicine as a home monitoring solution to manage the patients' health during and after the pandemic. Another opinion piece titled “Digital Covid credentials: an implementation process” by Nehme et al. reflects on the acceptance of digital covid credentials to ensure the implementation of adequate safeguards. It urges that the digital aspects of published information within a trust framework can be very useful to certify an individual's most recent Covid related status.

A policy and practice review on “A crisis-responsive framework for medical device development applied to the Covid-19 pandemic” by Antonini et al. urges for a crisis-responsive design framework to assist with product development, under pandemic conditions. The review emphasizes on stakeholder engagement, needs assessment, rapid manufacturing, and modified product testing to enable accelerated development of healthcare products. The study highlighted the use of crisis-responsive framework in a case study of face shield design and production for a large US academic hospital. A mini-review on “Covid-19 prognostic models: A pro-con debate for machine learning vs. traditional statistics” by Hindawi et al. explored the possibilities of data science to access open datasets, tutorials, programming languages, and hardware to create mathematical models to address the Covid-19 pandemic. It advocates the use of data science models to trace the impact of the virus on population and individuals for further diagnostic, prognostic, and epidemiological observations and analysis. It also had a comparative analysis between the classical statistics and machine learning techniques used for predicting covid outcomes.

The perspective by Fan et al. on “Factors to consider in the use of vital signs wearables to minimize contact with stable Covid-19 patients: experience of its implementation during the pandemic” cited reasons for choosing vital signs wearables for the purpose of reducing patient contact and preserving personal protective equipment. The study provides an overview of the factors needs to be considered while implementing vital signs wearable solutions in healthcare institutions. An original research study by Zhang et al. titled –“A real time portable IoT system for Telework tracking” validates the idea of using an Internet of Things (IoT) system to monitor the working status in real-time so as to record the working pattern and nudge the user to have a behavior change. The constructed shallow convolutional neural network (CNN) in the study helps to recognize the working status from a common working routine. The method adopted in the study is useful to the workers wellness during the Covid pandemic and also have a significant contribution in dealing with post-pandemic realities.

The perspective on “Integrated Care in the era of Covid-19: Turing vision into reality with digital health” by Kouroubali et al. looks into how digital health has been accepted as care platform across the globe to deal with the pandemic challenges. The perspective highlights the importance of digital health as an integrated care to support sharing and reusing of healthcare data for prevention, prediction and disease management. The study also addresses the political and social barriers and how to overcome them to achieve integrated care in practice. The perspective by Ramadi and Srinivasan on “Pre-emptive innovation infrastructure for medical emergencies: accelerating healthcare innovation in the wake of a global pandemic” proposes a pre-emptive innovation infrastructure incorporating in-house hospital innovation teams, consortia-based assembly of expertise, and novel funding mechanisms to combat health emergencies like Covid. The perspective talks about a framework to improve ongoing innovation and infrastructure for healthcare agencies by leveraging the strengths of academic, medical, government, and industrial institutions. The research study titled—“Covid-19 in Brazil—preliminary analysis of response supported by Artificial Intelligence in Municipalities” by Morales et al. emphasizes on the use of Artificial Intelligence to empower telehealth to increase coordinated patient access to health system during covid pandemic. The study describes a case report analyzing the use of Laura Digital Emergency Room as AI-powered telehealth platform in three different cities of Brazil. The study says that the implementation of an AI-powered telehealth will increase the access to healthcare services amid the unprecedented impact of Covid. The study urges for efforts to sustain affordable and scalable solutions to leverage value in health care systems in the context of middle and low income countries.

The research study by Kyriacou et al. on “Operating an eHealth System for pre-hospital and emergence health care support in light of Covid-19” talks about creating an electronic system (eEmergency System) in order to support, improve, and help the procedure of handling emergence calls. The study while quoting examples of case studies from Cyprus, focuses on developing an electronic system to support ambulance fleet handling, emergency call evaluation, triage procedure, and the improvement of communication between the call center and the ambulance vehicles. The system was further expanded during the Covid time in order to support the handling of patients infected with the new virus. The brief research report on “combinational analysis of phenotypic and clinical risk factors associated with hospitalized Covid-19 patients” by Das et al. is a follow-up study of using genomic data to identify a potential role of calcium and lipid homeostasis in severe Covid cases. The study attempts to identify similar combinations of features (disease signatures) associated with severe disease in a separate patient population with purely clinical and phenotypic data. The study used a Precision Life Combinational Analytics Platform to analyze features derived from de-identified health records in the United Health Group Covid-19 Data Suite. The study found several disease signatures where lower levels of lipids were found co-occurring with lower levels of serum calcium and leukocytes. The study looked into how these signatures are attributed to similar mechanisms linking calcium and lipid signaling where changes in cellular lipid levels during inflammation and infection affect calcium signaling in host cells. The study demonstrates that combinational analysis can identify disease signatures associated with the risk of developing severe Covid-19 separately from genomic or clinical data in different populations.

Another research study on “digital contact tracing against Covid-19 in Europe: current features and ongoing developments” by Blasimme et al. examines the evolution of digital contract tracing in eight European countries and highlights that privacy and data protection are at the core of contact tracing applications in Europe, even though the countries differ in their technical protocols, and their capacity to utilize collected data beyond proximity tracing alone. The study reflects a shift from a strict interpretation of data minimization and purpose limitation toward a more expansive approach to digital contact tracing in Europe. The study by Moura et al. on “explainable machine learning for Covid-19 Pneumonia classification with texture-based features extraction in Chest Radiography” provides evidential grounds for understanding the distinctive Covid-19 radiographic texture features using supervised ensemble machine learning models based on trees through the interpretable Shapley Additive Explanations (SHAP) approach. The study used 2611 Covid-19 chest X-ray images and 2611 non-Covid-19 chest X-rays. After segmenting the lung in three zones, histogram normalization is applied to extract radiomic features. SHAP Recursive Feature Elimination with Cross-Validation is used to select features and Hyperparameter optimization of XGBoost and Random Forest ensemble tree models were applied through random search. The study showed a predominance of radiomic feature selection in the right lung, leading to the upper lung zone.

The policy and practice review titled–“toward a common performance and effectiveness terminology for digital proximity tracing applications” by Lueks et al. explores digital proximity tracing (DPT) for Sars-Cov-2 pandemic mitigation as a complex intervention to notify application users about possible risk exposures to infected persons. The review describes differences between performance and effectiveness measures and attempts to develop a terminology and classification system for DPT evaluation. It further discusses key aspects for critical assessments of integration of additional data measurements into DPT applications to facilitate understanding of performance and effectiveness of the applications. The research–“Retrospective analysis and forecasted economic impact of a virtual cardiac rehabilitation program in a third-party payer environment” by Harzand et al. forecasts the potential clinical and economic benefits of delivering a home-based virtual cardiac rehabilitation program based on a retrospective analysis of cardiac rehabilitation utilization and cost in a third party payer environment. The study performed a retrospective cohort study using insurance claims data from a large, third-party payer in the state of Pennsylvania.

Another study on “using machine learning to predict mortality for Covid-19 patients on day Zero in the ICT” by Jamshidi et al. urges for the use of machine learning based on typical laboratory results and clinical data registered on the day of ICU admission for the early prediction of severity of the disease in intensive care unit (ICU) patients to optimize treatment strategies. The study reveals that several machine learning algorithms, including Random Forest (RF), logistic regression, gradient boosting classifier, support vector machine classifier, and artificial neural network algorithms can be easily used to build classification models and the predictions can be studied by implementing the local interpretable model-agnostic explanation technique. The study by Gupta et al. on “Trends in Covid-19 publications: streamlining research using NLP and LDA” developed a comprehensive Latent Dirichlet Allocation (LDA) model with 25 topics using natural language processing (NLP) techniques on PubMed research articles about Covid. The study proposed a novel methodology to develop and visualize temporal trends to improvise existing online literature hubs. The last study in the topic collection as a review article by Kostkova et al. on “data and digital solutions to support surveillance strategies in the context of the Covid-19 pandemic” provides a comprehensive overview for the application of data and digital solutions to support surveillance strategies. It also talks about digital epidemiology, available data sources, and essential components of 21st Century digital surveillance, early warning and response, outbreak management, control and digital interventions in the context of Covid-19 pandemic and beyond.

The challenge in a post-covid world will be to validate the new technologies and innovations without assuming that they will work because they worked in an uncontrolled emergency situation during the pandemic. This requires new observations regarding the adoption and use of digital health tools and services to receive information and seek social and medical assistance and support in a post-covid world when again the people will have physical access to socio-economic infrastructure, facilities and services (3). The concern will be to look into how medium and low income countries will refocus on preventive healthcare in a post-covid world with diverse and massive population, with disproportionate health infrastructure that often impacts medical responses to health emergency situations (4). When the world will regain its feet in post-covid realties, the challenge will be to recalibrate public health strategies with the help of emerging digital healthcare technologies to strengthen preventive healthcare. Investigations are required to explore the role of government in incentivizing and encouraging healthcare industries to deal with the issues of post-covid safety and care. The integration of health technologies and innovations into health policies and response strategies could be one of the concerned areas of research in post-covid world. The understanding of comprehensive responses of countries that have been successful to deal with covid through the timely and effective deployment of health technologies to facilitate planning, surveillance, testing, contact tracing, quarantine, and clinical management will help other countries to improve their health strategies and response systems (5).

We hope that this Research Topic collection will give the audience some good readings to look into how healthcare tools and innovations facilitate and enable easy-to-use and affordable healthcare services by providing accessible interfaces connecting all the stakeholders of healthcare system in a holistic manner. If these healthcare innovations and technologies will be used sensibly and rationally, they have the potential to revolutionize healthcare access and delivery in a post-covid world especially for women, vulnerable and marginalized groups, and economically disadvantaged sections of the society. To effectively deal with the post-covid realities, integrating medical and healthcare services with health technologies and innovations should be the priority for healthcare providers and policy makers. To effectively deal with a Covid like situation in future, a mechanism is required to enable patient tracking, movement of citizens, identification of viral loads and disease clusters to strengthen monitoring and containment measures and these also need to discuss at various academic and research forums.

## Author Contributions

The author confirms being the sole contributor of this work and has approved it for publication.

## Conflict of Interest

The author declares that the research was conducted in the absence of any commercial or financial relationships that could be construed as a potential conflict of interest.

## Publisher's Note

All claims expressed in this article are solely those of the authors and do not necessarily represent those of their affiliated organizations, or those of the publisher, the editors and the reviewers. Any product that may be evaluated in this article, or claim that may be made by its manufacturer, is not guaranteed or endorsed by the publisher.
